# Biosafety regulatory frameworks in Kenya, Nigeria, Uganda and Sweden and their potential impact on international R&D collaborations

**DOI:** 10.1080/21645698.2023.2194221

**Published:** 2023-03-28

**Authors:** Isaac Ongu, Priscilla Olayide, Erik Alexandersson, Barbara Mugwanya Zawedde, Dennis Eriksson

**Affiliations:** a Science Foundation for Livelihoods and Development, Kampala, Uganda; b Department of Plant Protection Biology, Swedish University of Agricultural Sciences, Alnarp, Sweden; c Department of Plant Breeding, Swedish University of Agricultural Sciences, Alnarp, Sweden; d Mukono Zonal Agricultural Research and Development Research Institute, National Agricultural Research Organisation, Mukono, Uganda; e Department of Biotechnology, Inland Norway University of Applied Sciences, Hamar, Norway

**Keywords:** GMO, plant biotechnology, biosafety, regulatory framework, SSA

## Abstract

Gene technologies, such as transgenesis and new breeding techniques (NBTs), expand the toolbox for plant breeding. Many countries in Africa, however, have long been seen as “slow adopters” of gene technologies for several reasons, one being the lack of, or overly restrictive, biosafety regulatory frameworks. This is sometimes attributed to the influence of the precautionary-oriented EU biosafety policies. This study analyses and compares the biosafety regulatory frameworks and their implementation in Kenya, Nigeria and Uganda, and in the EU member state Sweden. The focus is on (1) the structure of the biosafety regulatory frameworks including the scope of the legislation, (2) the duration and cost of regulatory authorization for field trials with genetically modified (GM) plants, and (3) the regulatory approach to NBT products, i.e. to what extent NBT products are subject to the provisions of the biosafety regulatory framework. The data was collected through studying relevant legal and policy documents as well as interviewing regulatory officers and researchers in the respective countries. We found that the regulatory procedures in the selected countries are relatively straightforward, while the costs and duration may present a challenge. The regulatory approach to NBT products differ between the selected African countries and Sweden, the latter which follows EU regulations. The results are discussed in terms of the impact the regulatory developments in these four jurisdictions may have on international R&D collaborations involving the use of gene technologies and we also weigh the results against the common conception that Europe exerts a heavy influence on African countries in this technology field.

## Introduction

An efficient breeding program for major locally or regionally important staple crops is an essential component for a sustainable increase in agricultural output ^1^. To this end, recombinant DNA technology, sometimes referred to as transgenesis or genetic modification (GM), has been exploited in plant breeding for nearly three decades, delivering benefits in terms of pesticide reduction,^2^ crops with an improved nutritional proﬁle and with fewer health concerns^3^ and crops that provide environmentally sustainable industrial raw materials. New breeding techniques (NBTs) are a set of more recently developed gene technologies or concepts, such as site-directed nuclease (SDN) systems (commonly called gene editing), cisgenesis and intragenesis, epigenesis, and techniques that apply transgenesis in part of the process (reverse breeding) or part of the plant (grafting),^4^ for which regulatory approaches are emerging, providing clarity on whether they are covered by biosafety regulations. For a detailed technical description of NBTs, we refer to Hartung and Schiemann (2016).^5^

The Cartagena Protocol on Biosafety (CPB) to the Convention on Biological Diversity (CBD) has often served as a reference for countries to develop biosafety legislation for the products of gene technologies,^6^ as this international agreement addresses the need to protect human health and the environment from the possible adverse effects from transboundary movement of the products of modern biotechnology.^7^ Kenya, Nigeria, Uganda and the European Union (EU) have all ratified the CPB, and this forms the basis for risk assessment and risk management of genetically modified organisms (GMOs) in the selected SSA countries. The regulation of GMOs in the EU predates the CBD (and the CPB) and over the years an elaborate regulatory framework has developed that differs from that of the CPB in certain aspects, such as the definition of GMO which in the EU legislation is broader than that of the CPB.^8^

**Figure 1.**
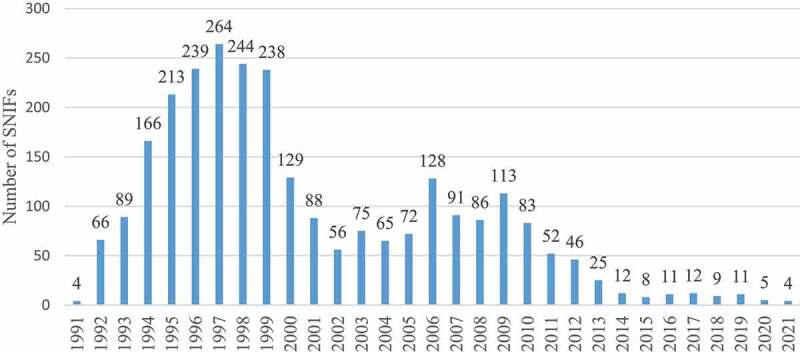
Number of summary notifications (summary notification information format, SNIF) from EU member states to the European commission about field trials with genetically modified plants in the years 1991–2021. Information retrieved from https://webgate.ec.europa.eu/fip/GMO_Registers/GMO_Part_B_Plants.php.

The EU has in the past two decades been notoriously restrictive with authorizing the cultivation of GM plants. The only agricultural crop event that has been authorized and cultivated to a large extent (the insect-resistant maize event MON810) is currently pending renewal of authorization to place on the market.^9^ Apart from that, only a few ornamental plant species are authorized.^10^ As a result of this lack of market, GM crop field trials in the EU have dropped dramatically. Also, a wave of destructions of GM field trials by activists has significantly contributed to this drop.^11^ The number of summary notifications (SNIFs; summary notification information format) from the EU member states to the European Commission peaked in the late 1990s but was reduced to nearly nothing from 2012 to 2013 and onwards (Fig. 1). Sweden is one of the very few countries in Europe that resists this trend and continues with GM crop field trials.^12^

In Africa, some countries that enacted biosafety laws are not yet utilizing gene technology while others have had field trials and are also beginning to authorize GM crops for cultivation.^13^ Beyond South Africa, which has cultivated GM cotton, soybean, and maize for over two decades,^14^ the most recent commercialization approvals in 2019 have been Bt cotton in Kenya and pod borer resistant cowpea in Nigeria. Other approvals for commercial cultivation of Bt cotton are in Ethiopia, Malawi, Sudan and Eswatini.^13^ Additional field trials are continuing (Table 1).^14,15^ Despite several field trials and commercial release authorizations though, African countries continue to carry a reputation of being “slow adopters” of GM technology.

**Table 1.** Approvals for GM crop field trials, commercial release and importations of GM products for food and feed use in Kenya, Nigeria, Uganda, and the EU (with a note on GM crop field trials in Sweden).

Country	Permit for field trial/confined field trial	Approval for cultivation (environmental release)	Approvals to import for food or feed use
Kenya	14 (Water efficiency in Maize, 2010; Cassava Mosaic Disease resistance in cassava, 2011; Pro Vitamin A enhancement in cassava,2011; Pro vitamin A, protein quality, Iron &Zinc enhancements in cassava, 2011; Resistance to Cassava Brown Streak Virus in cassava, 2012; Insect resistance in Maize, 2012; Cassava Brown Steak Disease resistance in cassava, 2013; Pink color stability in Gypsophila, 2013; Cassava Mosaic and Cassava Brown Streak Disease resistance in cassava, 2014; Sweet Potato virus resistance in Sweet potato, 2014; Provitamin A, Iron &Zinc enhancements in Sorghum, 2015; Pest and drought resistance in maize, 2015; Banana Xanthomonas wilt in banana, 2016; Late Blight resistance in potato, 2022) *Source: http://ke.biosafetyclearinghouse.net/approvedcft.shtml*	3 (Insect resistance in Maize for NPT and additional data collection, 2015; Insect resistance in cotton for NPT, 2016; Virus Resistant Cassava 2021) *Sources*: *https://s3.amazonaws.com/bch.webfiles/4eb8/cbed/87bd82fc839f0060b7780c3e?AWSAccessKeyId=AKIAT3JJQDEDD6EIMETR&Expires=1644410932&response-content-disposition=inline%3B%20filename%3D%22public_notice.pdf%22&response-content-type=application%2Fpdf&Signature=cY95LmqwqVPPpUUPyHGF0o2Ujco%3D* *http://ke.biosafetyclearinghouse.net/cotton_application.pdf* *https://www.biosafetykenya.go.ke/images/NBA-Board-Approves-application-for-Genetically-Modified-Cassava-FINAL.pdf*	28 approvals of importations of maize and soybean with insect resistance/herbicides tolerance traits for humanitarian purpose between 2011 and 2012 *Source: http://ke.biosafetyclearinghouse.net/importandtransit.shtml*
Nigeria	4 (Elevated levels of Zinc and Iron in cassava roots, 2019; Drought tolerance and Insect resistance in Maize, 2019; Insect resistance in Cow pea; Late blight resistance in potato, 2022) *Source: https://nbmagov.ng/permits-granted/* *https://nbma.gov.ng/permit-decision-document-issued-to-national-root-crops-research-institute-umudike/*	1 (Insect resistant cowpea, 2019) *Source: https://nbmagov.ng/permits-granted/*	9 permits (soybean and maize combined for feed food and processing) wheat (2022) Note: some permits had over three events approved at once *Source: https://nbmagov.ng/permits-granted/*)
Uganda	8 (Black Sigatoka resistance in bananas, 2006; Herbicide tolerance & insect resistance in cotton, 2007; Bacterial wilt resistance in bananas, 2010; Drought tolerance in maize, 2010; Cassava Brown Streak Disease resistance in cassava, 2010; Insect resistance in maize, 2012; Virus resistance in sweet potato, 2013; Drought tolerance and Insect resistance (stack) in maize, 2015; Late Blight Resistance in potato, 2015;Herbicides tolerance in Soybean, 2016) *Source: Zawedde et al., 2018*	None	Products containing genetically modified foods available in Uganda markets *Source*: *http://nilepost.co.ug/2018/05/22/the-dark-secrets-behind-the-gmos-war-in-uganda/*
EU,Sweden	Three EU member states had field trials in 2021 (Belgium, Spain, Sweden). Sweden approved in the period 1991–2021 a total of 146 field trials with GM crops *Source: Eriksson et al., 2018b and* ^a^	-MON 810 approved in 1998, but banned by 17 member states and two territories. Cultivated currently in Spain and Portugal. Renewal of cultivation permit pending -Amflora by BASF, approved in 2010, approval annulled in court in 2013 after the event had been withdrawn by BASF.	- There are currently 89 food/feed/other products/events listed as registered (authorized) for sale in the European Union (15 cotton, 40 maize, 7 oilseed rape, 26 soybean, 1 sugar beet). - There are 14 pending product/event applications (5 maize, 1 soybean, 4 cotton, 4 oilseed rape). *Source: https://webgate.ec.europa.eu/dyna/gm_register/index_en.cfm*

^a.^ https://jordbruksverket.se/vaxter/odling/gmo-genetiskt-modifierade-organismer/om-gmo.

The construction and implementation of a biosafety regulatory framework has in many African countries mimicked the precautionary approach of the EU rather than the more liberal approach of the United States.^16^ Several reasons are mentioned for the potential influence of the EU on African biosafety policies, such as the bilateral and multilateral foreign aid/assistance, advocacy campaigns from international (often Europe-based) NGOs, the risk of losing agricultural export opportunities, with farm exports to Europe being six times larger than that to the United States, or a total of 40% of all SSA’s agricultural exports,^17,18^ and the stronger cultural ties to Europe of the political elite in many African countries.^18^ Recent developments however, with regulatory approvals in certain SSA countries as mentioned above, indicate that the EU influence may no longer be as strong as sometimes suggested. It is therefore not clear whether the biosafety regulatory situation in these countries present any obstacles for research and development (R&D) collaborations between African and EU countries that involve the use of gene technologies. Further insight into these interactions is needed to guide policy developments and research funding priorities.

## Objectives

This study compares the biosafety regulatory structure, scope, practices and implementation in Kenya, Nigeria, Uganda, and the EU member state Sweden. The specific objectives are to describe and compare their respective biosafety regulatory environments for gene technology research and development (R&D) in terms of (1) the structure of the biosafety regulatory frameworks, including the definition of regulated organisms in the legislations, (2) the duration and cost of the approval procedure for field trials with GM plants, and (3) the current or proposed regulatory approach to NBTs. The purpose is both to provide an overview of the biosafety regulatory environments in these countries and to investigate whether or not these respective environments may present any obstacle to international R&D collaborations involving gene technology activities. The scope of the study is limited to (1) the four mentioned jurisdictions (for Sweden, this includes any applicable EU law), (2) R&D activities but not commercial applications, (3) biosafety regulations applicable to the products of gene technologies, such as those based on the CPB, or the EU Directive 2001/18/EC.

## Methodology

This study is based on published scientific and legal literature as well as policy and legal documents from the national competent authorities (NCAs) of the selected countries, and on interviews with researchers and regulatory officers in the selected countries. Policy and legal documents reviewed include legislative documents (in the EU: Directives, Regulations and other secondary legal acts), guidelines governing the conducting of GM crops field trials, policy decision documents^19^ and publications, applications for field research, and approval documents. The documents were obtained from the public websites of biosafety agencies of Uganda,^20^ Kenya^1,21^ and Nigeria,^22^ and of the EU^2,3^ and Sweden.^23^ Additional relevant policy documents reviewed were the CBD and the CPB.

Interviews with researchers applying gene technology in plants were conducted between September and October 2019 and interviews with representatives from NCAs in the selected SSA countries were conducted via online video meetings in August 2020. The individuals interviewed were principal investigators or field trial managers from the following institutes: Swedish University of Agricultural Sciences (SLU), Kenya Agricultural Research and Livestock Organisation (KALRO), National Agricultural Research Organization (NARO) in Uganda, and National Root Crops Research Institute in Nigeria; and regulatory heads of National Biosafety Management Agency (NBMA) of Nigeria, National Biosafety Authority (NBA) of Kenya and National Biosafety Committee (NBC) of Uganda.

For Kenya, Nigeria, and Uganda, “confined field trial” (CFT) means, “*a field trial of a genetically modified plant that has not been approved for general, release, measure for reproductive isolation and material confinement are enforced to confine the experimental plant and genes to the trial site*,” whereas “environmental field release” means cultivation for the purpose of production (commercial, or subsistence). The NCA in Sweden does not operate with measures of confinement for GM crop field trials, apart from distance to nearby fields as part of co-existence measures to conventional and organic farming. Other EU member states do operate with measures of confinement; however, these are not considered in this study.

## Results

### Structure of the Biosafety Regulatory Frameworks

A timeline of the development of the biosafety regulatory frameworks in the EU, Kenya, Nigeria, and Uganda is presented in Table 2.

**Table 2.** Timeline of the legal and policy developments for biosafety in the EU, Kenya, Nigeria, and Uganda.

Year	EU		Kenya		Nigeria	Uganda	
2020			Guidelines for NBTs awaiting approval by NBA board		Guidelines on NBTs under formulation	Guidelines on NBTs under formulation	
2019					NBMA amended to include NBTs	Biosafety law amended and renamed GERA and passed, NEMA Act 2019	
2018	ECJ ruling on mutagenesis						
2017					Updated National Biosafety Policy passed	National Biosafety law passed by Parliament	
2015	Directive (EU) 2015/412		Environmental Management and Coordination Act (EMCA) amended		Biosafety law, NBMA Act established	Plant Protection and Health Act	
2014						Plant Variety Protection Act	
2013					Rejected Biosafety Bill returned to Parliament, Seeds Act, Seed Council		
2012			Cabinet ban on importation of GM foods, KEPHIS, Seeds and Plant Vty Act			National Biotechnology and Biosafety Bill tabled	
2011					Biosafety Bill passed in Parliament, rejected by President		
2009	2009/41/EC		Biosafety Act creating NBA established				
2008						Biotechnology and Biosafety Policy passed	
2007					Environment Agency, NESREA formed		
2006			National Biotechnology Policy established		National Biosafety Framework developed	Seed and Plant Act	
2003	Regulation 1829/2003; Regulation 1830/2003				Ratified Cartagena Protocol		
2002	EFSA created; Approved CPB		Ratified CPB				
2001	Directive 2001/18/EC repealing 90/220/EEC					Ratified CPB	
2000	Signed CPB		Signed CPB		Signed Cartagena Protocol	Signed CPB	
1999			NEMA established by EMCA				
1996						National Biosafety Committee established	
1995						NEMA established	
1994			Ratified CBD		Developed National Biosafety Guideline, Ratified CBD		
1993	Approved CBD					Ratified CBD	
1992	Signed CBD		Signed CBD		Signed CBD	Signed CBD	
1991							
1990	Directive 90/219/EEC;90/220/EEC					UNCST Act establishing UNCST	
Key							
Global Policy		Domestic Environment laws and regulations			Biosafety laws and regulations	Seed and Plant Variety laws	

#### Kenya

Kenya adopted a Biosafety Development Policy in 2006 and enacted the Biosafety Act in 2009. The Act established the National Biosafety Authority (NBA), which is the competent authority that administers biosafety regulations and guidelines in Kenya. The NBA is also a National Focal Point for the CPB and the Biosafety Clearing House. NBA is governed by a board comprised of nine members: a chairman (who must be a scientist), representations from five Government ministries (Agriculture, Environment, Science & Technology, Finance, and Health), two independent experts in biological, environment or social sciences, and Chief Executive Officer who is an ex-officio member. The NBA is housed in the Ministry responsible for Science and Technology. The biosafety implementing regulations of the 2009 Biosafety Act consist of 1) contained use regulations that give directions on how to conduct research in the laboratory, greenhouse, and confined field trials; 2) environmental release regulations for placing on the market; 3) import, export and transit regulations; and 4) labeling regulations. The National Environmental Management Authority (NEMA) and Kenya Plant Health Inspectorate Service (KEPHIS) also play roles in the biosafety regulatory process. Institutions involved in Biotechnology research must have Institutional Biosafety Committee (IBC) to guide compliance with the biosafety regulations. The GMO definition in the Kenya’s Biosafety Act, National Biosafety Act (2009) is defined as, “*any organism that possesses a novel combination of genetic material obtained through the use of modern biotechnology techniques*.”^4,24^

#### Nigeria

Regulations of GMOs in Nigeria started in 1994 with the development of a National Biosafety Guideline. These were developed to authorize CFTs prior to enactment of a law. A National Biosafety Committee (NBC) was created in 2000 and it played a critical role in the process of developing the law. The National Biosafety Management Agency Act was enacted in 2015 and it established the National Biosafety Management Agency (NBMA). NBMA is the competent authority for biosafety regulations including approvals for applications of transgenic and genome edited crops. The NBMA establishes the NBC to review the application and provide scientific opinion to the NBMA. NBMA may appoint technical experts or institute a National Biosafety Technical committee to provide technical advisory support toward the decision-making process of the NBMA. NBMA is housed in the ministry responsible for Environment. The NBC composition includes representatives from the Environmental Management Agency and Federal Ministry of Agriculture and Rural Development. Memorandums of understanding exist between the NBMA and these regulatory agencies, which ensures coordination among the agencies. Institutions involved in Biotechnology research have an Institutional Biosafety Committee (IBC) to guide compliance with the biosafety regulations. The GMO definition in the Nigeria’s Biosafety Act (2015) is, as in Kenya, based on the LMO definition of the CPB. The regulation and its implementation of GMOs is prescribed in the National Biosafety Regulations of 2017.

#### Uganda

The current biosafety framework is based on the Uganda National Council for Science and Technology (UNCST) Act of 1990. UNCST established the NBC as early as 1996 as a technical committee to handle applications for contained and confined research that require biosafety approval. The NBC developed guidelines on contained use and confined field trials of GM plants. A National Biotechnology and Biosafety Policy was adopted in 2008. Thereafter, a process commenced to develop a biosafety law that was passed by Parliament in 2017 as the National Biosafety Act, and later revised and passed in 2018 as the Genetic Engineering Regulatory Act.^25^ For this Act to come into force, it must be assented to by the President; this final step is still pending to date. In absence of this law, UNCST through NBC continues to approve and oversee all GMO-related R&D activities in Uganda. The amended NEMA Act, 2019 also in section 63 on Management of GMOs provides two clauses that state, *“NEMA may, in consultation with the relevant lead agency, issue guidelines and prescribe measures—(a) for the protection of the environment and management of risks to human health from the development, access, use and transfer of GMOs; and (b) for liability and redress in relation to GMOs.”* Conservation of biological resources in situ and ex situ, and access to the genetic resources of Uganda are covered under the amended NEMA Act as well. Other regulatory agencies involved in biosafety regulations include: the Department of Crop Inspection and Certification under the ministry responsible for Agriculture; and the Uganda National Bureau of Standards under the ministry responsible for Trade that guides food safety standards. The Seeds and Plant Act (Seeds & Plant Act, 2007) has a clause that states *“genetically modified seeds will be regulated in accordance with the Uganda National Council of Science and Technology Act or any relevant law.”* The GMO definition adopted in the National Biotechnology and Biosafety Policy (2008) is *“an organism in which a gene or genes has/have been artificially inserted.”*

#### EU

The regulatory framework for field release of GMOs in the EU was adopted in 1990 with EU Council Directive 90/220/EEC on the deliberate release into the environment of GMO. This directive provided for a rather decentralized authorization procedure but was revised in the late 1990s and resulted in the current legislation including mainly Directive 2001/18/EC on the deliberate release of GMOs into the environment, Regulation (EC) 1829/2003 on genetically modified food and feed, and Regulation (EC) 1830/2003 on traceability and labeling of GMOs. The current EU GMO regulatory framework entails a centralized case-by-case risk assessment procedure by the European Food Safety Authority (EFSA) and harmonized decisions that are by default valid in all member states. There is however plenty of scope for national decisions related to the cultivation of GMOs. Apart from a safeguard clause (Art 23, Dir 2001/18/EC), by which member states may provisionally restrict or prohibit the use and/or sale of GMO on its territory, also Directive (EU) 2015/412 allows member states to “opt out,” that is, to restrict or prohibit cultivation of GMOs in their territory based on a number of considerations apart from established concern of risk. Despite a harmonized framework among the EU member states with respect to the scope and definition, risk assessment, authorization, monitoring and labeling, the individual member states have a high degree of sovereignty in deciding on details related to field trials and co-existence measures. For field trials with GM crops, the national authorities in the country where the release is planned has the mandate to decide on its authorization as well as setting the conditions for its implementation. This means that the possibility to do GM field trials may differ substantially between EU member states. The definition of a GMO, as stated in Dir 2001/18/EC (Art 2), is “*an organism, with the exception of human beings, in which the genetic material has been altered in a way that does not occur naturally by mating and/or natural recombination*.” This definition also covers the products of randomly induced mutagenesis and cell fusion, making it broader than the LMO definition of the CPB.

### Regulatory Authorization Procedure for GM Field Trials

#### Kenya

The application for GM field trials in Kenya is submitted to the NBA secretariat. The application, which must have a recommendation from the Institutional Biosafety Committee (IBC), is screened for administrative and technical completeness before the applicant is issued with an acknowledgment of receipt. The approval is done with the participation of the technical committee and two independent reviewers whose recommendations inform the final decision. In case of need by an applicant to import materials for research, the applicant is directed to KEPHIS for import permit. KEPHIS has a representative on the NBA technical committee. NBA’s “Guide to applicants on GMO application submission timelines”^26^ indicates that in circumstances where an applicant is not asked to provide additional information, a decision on GMO application is made within 150 days from the date of acknowledgment of Receipt of application (Table 3). The countdown for decision-making period is paused whenever an applicant is required to provide additional information. The application costs for contained use, confined use, import, export, and transit of GMOs are provided in Table 3. NBA issues approval decisions for all GMO applications received quarterly. In the period 2010–2022, fourteen approvals for confined field trials (CFTs) have been issued, and three approvals for environmental release in 2015–2022 (Table 1). Environmental release referred to as “intentional introduction into the environment” in the NBA Act,^24^ implies introducing GMOs into the environment deliberately after confinement for the purpose of conducting variety registration trials to make them available to the public, such as for cultivation.

**Table 3.** Duration and costs related to the regulatory requirements for research activities with GMO in Kenya, Nigeria, Uganda, and Sweden. GMO, genetically modified organism; CFT, confined field trial.

	Kenya	Nigeria	Uganda	Sweden
Maximum time to decision on GM crop field trial application	150 days	260 days	90 days	120 days
Cost for GM crop field trial/CFT application	USD 1,395 (valid five years)	USD 9,360 Fast track: +USD 2,600	USD 1,800 for one site+ USD 300 per site (valid one year)	Approximately USD 5,370 (SEK 46,000) (valid five years)
Cost for renewal of permission for GM crop field trial/CFT	USD 233	USD 1,560 Fast track: +USD 3,900	USD 500	Approximately USD 3,090 (SEK 26,500)
Cost for inspection of GM crop field trial site	N/A	N/A	N/A	Approximately USD 455 annually (SEK 3,900)
Cost for import, export and transit of GMO	USD 465	USD 780 Fast track: +USD 130	Approximately USD 6 (UGX 20,000)	N/A (based on harmonized decision in the EU)

#### Nigeria

The application for GM field trials in Nigeria is similar to Kenya and Uganda, submitted to the Biosafety Agency, NBMA with an endorsement of the IBC. The IBC is either part of the local institution under which the trials are conducted, or an institution designated to review application before submission to the NBMA. An applicant should propose risk management measures in case of anticipated adverse effects. The local research institutes and trial sites are certified by the NBMA. The NBMA endorses and confirms receipt of application in writing within 21 days. The NBMA then may constitute a committee that reviews and recommends applications for considerations by the NBMA. Decision to approve or reject is made by the agency within 270 days after acknowledging receipt of the application. The application approval decision contains the terms and conditions for approval or justifications for rejection. Shipment into Nigeria of GM products and materials for research purpose is approved by the NBMA. The NBMA inspects the materials and required documents on arrival before permitting or rejecting it. After inspection, the applicant is issued a transit permit or transit denial. Issues requiring liability and redress conform to the Cartagena supplementary protocol on redress and liability to which Nigeria is signatory. The decision-making for approval of field trials in Nigeria can be fast-tracked at an additional cost to the applicant. The period within which a fast-tracked decision should be made is not expressly stated. At the time of the study, no applicant had utilized this provision. The application costs for contained use, confined use, import, export, and transit of GMOs are provided in Table 3. In 2019, three approvals for CFTs were issued and one approval for environmental release (Table 1).

#### Uganda

Application for field trials with GMOs in Uganda is guided by the NBC’s “National Guidelines for Field Trials of Genetically Engineered plants.” An applicant is required to first submit the application to the Institutional Biosafety Committee (IBC). The IBC reviews and provides the recommendations to the applicant, who then submits the application to the competent authority together with the IBC’s recommendations. The competent authority (UNCST) reviews the application within ten days for “completeness.” The applicant is notified on an incomplete application, and it is the applicant’s responsibility to provide missing information before the application is forwarded to NBC for review. NBC reviews and decides on the application within 90 working days, after acknowledging date of receipt of a complete application (Table 3). When approved, the trial site is inspected before planting.^27^ In case an applicant needs to import GM plants into the country for research purpose, the application for the import permit is submitted for approval to the Department of Crop Inspection and Certification under the Ministry of Agriculture. The application should include a letter confirming that there is no objection from the NBC, and a phytosanitary clearance from the country of origin. A crop inspector from the Ministry of Agriculture does the inspection at the point of entry. The application costs for contained use, confined use, import, export, and transit of GMOs are provided in Table 3. In 2006–2016, a total of eight approvals have been issued for CFTs, and none for environmental release so far.

#### EU

In the EU, an application for a field trial with a GMO is submitted through the NCA of the member state. In Sweden, the NCA for GMO cultivation is the Swedish Board of Agriculture (SBA). It takes a maximum of four months between application submission and decision (Table 3). The NCA publishes a summary for public comment and the application is sent for referral to a number of public authorities, universities and other organizations in Sweden. The NCA is also required to supply the European Commission with Assessment reports and notification under the Summary Notification Information Format (SNIF). The application costs in Sweden for contained use, confined use, import, export, and transit of GMOs are provided in Table 3. As there is plenty of leeway for individual EU member states to decide on authorizations, application costs, monitoring, co-existence, and other relevant aspects of GM field trials, it is difficult to say something generally applicable in the EU about the prospects of getting authorization and the costs involved. Sweden can be characterized as one of the more permissive EU member states in terms of approving applications for GM field trials,^12^ whereas it is much more difficult, if not impossible, in some other member states. In 1991–2021, a total of 146 decisions were taken by the SBA to authorize field trials with many different GM crops and model plants in Sweden^5^ (Table 1).

### Regulatory Approach to New Breeding Techniques

#### Kenya

Kenya’s NBA had drafted guidelines for gene edited crops, awaiting approval by the NBA board at the time of the interview in August 2020. In February 2022, the NBA published the guidelines for determining the regulatory process of genome edited organisms and products in Kenya. This publication described genome edited products that are regulated under the Biosafety Act. According to this document, genome edited products achieved through deletions/knockouts in which there is no insertion of foreign genetic material in the end-product and products whose inserted foreign genetic material cannot be detected are not regulated under the Biosafety Act. As of February 2022, there were no field trial approvals of crops developed through NBTs in Kenya, but six applications involving gene editing have been approved for contained use. These include pro-vitamin A enhancement in cassava, fusarium wilt & black Sigatoka resistance in banana, early flowering in cassava, striga resistance in sorghum, early flowering in grass pea, nematode resistance in banana, pro-vitamin A enhancement in yams, and virus resistance in cassava (NBA, 2021). Prior to the new regulatory guidelines on gene editing, gene-edited crops were regulated as a GMO.

#### Nigeria

Following the amendment in 2019 of the NBMA Act of 2015, Nigeria started to formulate guidelines for gene-edited crops. The NBMA Act of 2015 as amended in 2019 has a new section 25A, “Application of gene drive, gene editing and synthetic biology.” The amendment emphasizes the mandate of NBMA as the only authority to regulate gene-edited crops and defines gene editing as “*a type of genetic engineering in which DNA is inserted, deleted, modified or replaced in the genome of a living organism*.” The interview with Nigeria’s regulatory official confirmed that Nigeria amended its GMO law to include the definition of gene editing to ensure clarity. The draft guidelines for regulating gene editing in Nigeria was pending final approval at the time of the interview in August 2020 and no gene-edited crop was approved for field trial at the time according to the regulatory official interviewed. The guideline that was later published in April 2021 as the National Guideline for the Regulation of Gene Editing allows gene-edited products without transgenes to be treated as conventional products. By including gene editing in the NBMA Act, these applications and products are covered by the biosafety regulations but may not have to go through the full risk assessment and review process.

#### Uganda

Uganda continues to use UNCST Act of 1990 that mandates UNCST to regulate all research in the country. By the time of the interview, UNCST through NBC were drafting guidelines for gene-edited crops. According to the regulatory officer interviewed, Uganda will, like Kenya and Nigeria, exempt products of NBTs without transgenes from undergoing the GMO assessment. Uganda as well did not yet have any field trials with gene-edited crops.

#### EU

The legal status of the products of NBTs has been under discussion for several years.^28^ There is an ongoing process to shape the EU approach to NBTs, affected by a ruling on mutagenesis in 2018 by the Court of Justice of the EU (CJEU)^29^ and a recent report on new genomic techniques (NGTs) by the European Commission.^6^ In the meantime, there are field trials with genome-edited plants, including potato and aspen in Sweden, potato in the Netherlands, maize in Belgium and tobacco in Spain, but they are to our knowledge all carried out with permit under the GMO legislation.^30^ In 2018, a Court of Justice of the European Union (CJEU) ruling stated that the products of new methods of mutagenesis should be subject to the provisions of the GMO regulatory framework, and not exempted from regulation as the products of conventional methods of mutagenesis (i.e., randomly induced) are. This ruling was limited in scope however, and there is still legal uncertainty for the products of other NBTs.^31^ In this context, it is important to point out that a plant mutated through the application of gene editing (e.g., CRISPR/Cas) may not necessarily be considered a product of mutagenesis, and hence a GMO, under the EU law. On request from the Council of the EU, the European Commission (EC) therefore prepared a report on the status of NGTs. For the purpose of the report, NGTs are defined as techniques capable to change the genetic material of an organism and that have emerged or have been developed since 2001, and the report concluded that the products of all NGTs should be regulated as GMOs.^7^ This has triggered a process of legal reform in the EU. By the second quarter of 2023, EC is expected to present a proposal for a legal framework for plants obtained by targeted mutagenesis and cisgenesis and for their food and feed products.^32^

## Discussion

The minimum required elements of a functional biosafety regulatory framework include (1) the laws, regulations and guidelines, and (2) an institutional structure and capacity for implementation, with experts capable of reviewing applications and making informed decisions including on potentials risks, public participation strategies, and post-authorization measures such as monitoring and traceability.^33^ All these elements of an established biosafety regulatory framework are present in Kenya, Nigeria, Uganda, and all the EU countries. Having recognized the existence of the basic structure of biosafety regulatory frameworks across the countries of study in terms of administrative institutions, laws and guidelines, we show that at country levels the practices by the public authorities as in Nigeria where the MoU^34^ among agencies enables binding decisions making, and the political environment as in Kenya where cabinet “approves,”^35^ play a great role in final decisions making especially in determining environmental release after confined field trials are successfully completed. We look at whether the purported influence by the EU is recognizable in the regulatory scope, and nature of decisions made. We further look at how emerging technologies like new breeding techniques are being treated in the SSA countries of choice in comparison to what is happening in the EU, and the implications to collaborations on Research and Development between the EU and SSA countries. We also point out in recommendation how countries could learn and perhaps adopt practices that have worked elsewhere. These insights could help those who would want to partner with African research institutions appreciate that existing institutional and regulatory frameworks alone may not necessarily tell how the public authorities implement such laws and guidelines, and how the political environment remain key in decision-making process, and initiatives to continuously provide information that inform evidence-based decision-making remain vital.

### Regulatory Authorization Procedure for GM Field Trials

It has long been argued that the high regulatory costs present an obstacle to the development of plant gene technologies in African countries.^36,37^ Smyth and Zepeda showed through case studies that the time and cost for the transformation research phase, including field trials, made the investment too high for research institutes in many countries in Africa and were only possible through regional and international cooperation.^38^ Field trials with GM plants (CFTs as in SSA countries where trials are fenced off and guarded all the time or open-air as it is in Sweden where trials are in an open field and not guarded by security personnel) have been conducted in all countries in our study and the number of approvals is listed in Table 1. Kenya has the highest number of CFT approvals (twelve trials in 2010–2016), followed by Uganda (eight trials in 2006–2016) and Nigeria (three trials in 2019). However, the regulatory practices in relation to these field trials vary between the countries in terms of duration and cost of the approval process, and this may have an impact on the ability for research groups to carry out the research. The maximum number of days prescribed in the regulations and guidelines for decision on a field trial application vary from 90 days in Uganda, 150 days in Kenya, to 270 days in Nigeria, whereas in Sweden the decision is taken within 120 days. From the interviews, it was mentioned that there were no delays emanating from any regulatory inefficiency in the selected countries in approving applications for field trials. However, the duration can be exceeded if an applicant is required to provide additional information, as the days taken by an applicant to return with information is not made part of the mandatory maximum duration. The practice is for regulators to stop counting the days as they wait for any additional document needed from an applicant. A detail that may delay the process is that applicants for GM research in Kenya, Nigeria and Uganda require recommendations from the accredited Institutional Biosafety Committee (IBC) to be considered for review and eventual approval. We do not have any data on how long this takes, however it is something that needs to be considered for researchers’ activities with GM plant material in these countries.

Regarding the approval process for GM field trials, the interviews revealed timely approvals. Major delays are at the environmental release and placing on the market stages.Nigeria’s approval framework provides for extra charged for “expedited option” for any applicant who may require to fast-track an application review process whenever they deem necessary. According -to the applicant interviewed, there was never been a need to pay for “expedited review” for any of their applications. John Komen and Leena Tiipathi (2020),^39^ attributes the generally speedy process in reviewing GM crop applications to the greater emphasis being put on anticipated benefits taking advantage of experiences and data from other countries. Timeliness in approving applications for field trials remain important, and there was no case for unnecessary delays in field trials approvals in Kenya, Nigeria and Uganda.

Nigeria stands out with the highest costs for field trial applications, at nearly twice the cost as in Sweden and several times higher than in Kenya and Uganda. The impact of this difference is much exacerbated when considering the economic state of the countries as reflected by the gross domestic product (GDP) per capita, which in 2020 were USD 1,838 for Kenya, USD 2,097 for Nigeria, USD 817 for Uganda, and USD 52,259 for Sweden.^8^ These costs may thus, in particular for Nigeria, take a heavy toll on the budgets for research projects (in part depending on the source of funding). The total compliance costs vary based on the crop, trait and country.^40^ The exact costs for standard operating procedures to ensure compliance are beyond the scope of this study; however, it is should be noted that high compliance cost may reduce investments in biosafety-regulated products,^38^ and may also have a considerable impact on the possibility to carry out research collaborations. The high cost of developing a GM product is undesirably high for public research institutes who are major developers of new crop varieties in Africa.^38^

Despite decisions made by established or recognized biosafety authorities during the field trial phase, environmental agencies in the three selected SSA countries have more influence in the decision-making processes especially in the approvals for environmental release, cultivation and placing on the market, and in several instances, cause delay in final approvals. This is prominent in Kenya where the NBA’s approval decision^41^ in respect to an application for environmental releases, cultivation and placing on the market is subject to the applicant submitting an Environmental and Social Impact Assessment (ESIA) to NEMA, in addition to complying with other relevant laws and policies. Kenya explicitly included introduction of GMOs in its 2015 amended Environmental Management and Coordination Act^40^ among events that must undergo Environmental Impact Assessment (EIA) study before it is allowed in the environment. In Nigeria, a formal Memorandum of Understanding (MoU) between the biosafety authority and other Government agencies like the National Environmental Standards and Regulations Enforcement Agency (NESREA) and National Agricultural Seed Council (NASC), and National Agricultural Quarantine Service (NAQS) in Nigeria has helped in harmonized general release/placing on the market decisions, as decisions made by the NBMA does not require further unusual scrutiny from other Government agencies. The Chief Executive Officer of NBMA as reported by the Guardian (2017),^34^ says, “*the MoU presented the various agencies the needed platform for synergy and opportunity to fast-track the management of issues of genetic modification in a way to safeguard the health of Nigerians and the environment.”*

The purpose of Nigeria’s MoU with other relevant Government Agencies could explain why approval decision by the NBMA is not limited to further approval for environmental safety as it is in Kenya where the NBA makes “limited release” approval requiring an applicant to separately fulfil environmental safety requirements. Kenya has also had practices where final decisions regarding GMOs are made by cabinet: ban on importation of GMOs^42;^ approval of GMOs for cultivation^35;^ and lifting of the ban on importation.^43^ In Uganda, the decision made by the NBC that has representation from all relevant regulators including NEMA, has been through consensus. This however, is yet to be tested during time for environmental release and placing on the market applications that is yet to happen in Uganda because Uganda’s current law’s mandate stops at confined field research. Kenya and Uganda too could consider having a formalized MoU between the Biosafety Authority and NEMA, and perhaps any other regulatory agency like KEPHIS in Kenya and Department of Crop Inspection and Certification in Uganda. The MoU would guarantee that the parties honor joint decisions in order to avoid unnecessary delays. Agreement or guidelines on how and when to collect particular sets of data, and how such collected data can be shared among the agencies for decision-making should be formalized to avoid unnecessary repetitions of the same process leading to delay in delivery of the final products to the intended beneficiaries. Though unverified, the transition from field trial to environmental release and finally placing on the market could impact immensely on the choice of country for collaboration in gene technology research.

### Regulatory Approaches To New Breeding Techniques: Constraint or Opportunity?

Fifteen years ago, the delays in preparing national biosafety regulations and guidelines were stated as a reason for the lack of progress in plant biotechnology in African countries.^36^ This situation has arguably been addressed to a large extent by now, as demonstrated by our examples from Kenya, Nigeria and Uganda. The present question is to what extent these biosafety frameworks are relevant for the more recent progress in plant gene technologies. Kenya, Nigeria and Uganda have all taken steps toward providing guidelines for gene-edited organisms. The tendency we have spotted is that these guidelines, if approved (as already in Nigeria and Kenya), could lead to a situation where gene-edited crops that do not carry transgenes are treated as conventionally bred crops. Kenya’s regulatory approach toward gene editing shows that Kenya’s guideline will allow for case-by-case assessments focusing on whether there is a novel gene present in the end product.^39^ A similar situation is developing in Uganda as well, according to our interviews with regulatory officers. This position of the regulatory authorities in Kenya, Nigeria, and Uganda differs from the current implementation of the EU GMO regulations as they are interpreted by the European Commission.^44–47^ Despite prevailing legal uncertainties that, among others, relate to the lack of definitions of key terms in the legislation, the EU authorities are currently adopting the policy that all products of NBTs (including gene editing) are classified and regulated as GMOs. The situation we have observed in Kenya, Nigeria and Uganda is therefore to some extent contrary to the usual impression that African countries follow in the footsteps of the EU. This position could see NBTs provide an opportunity for Africa to overcome legal and policy bottlenecks often associated with GMO legislation. As these policy and regulatory developments are still ongoing, it is still too early to estimate what effects they will have on R&D collaborations, but it is reasonable to expect that a regulatory framework that is permissive of safe applications of gene technological tools in plant R&D will facilitate, and even promote, the implementation of these tools. The CBD’s latest technical report^48^ on synthetic biology also recognizes the potential role of gene editing toward contributing to achieving several United Nation’s Sustainable Development Goals including Zero hunger. It, however, does not offer any binding position on how best to regulate gene-editing technologies other than recognizing “silo” efforts being made by individual countries. Gene-editing technique therefore provides Kenya, Nigeria, and Uganda with opportunities to address crop challenges like drought, pests and diseases, without the regulatory burden associated with GMOs, as well as attracting new collaborations that could have been discouraged by the previously stringent regulatory regime. Kenya already has six research activities on gene-editing under laboratory research.^49^

### Are the EU Biosafety Policies Influencing Those of the Selected SSA Countries?

It is often argued that the EU precautionary approach to gene technologies has exerted a strong influence on the corresponding policies in Africa. Paarlberg presents five arguments for this, including (1) the larger bilateral foreign aid from Europe as compared, for example, to that of the USA, (2) the technical assistance provided through the UNEP/GEF Global Project for Development of National Biosafety Frameworks, where European governments had considerable influence, (3) the advocacy campaigns of the mostly Europe-centered biotech-critical NGOs, (4) the commercial agricultural trade, which is (in 2010) six times larger than the exports to the USA, and (5) the cultural ties, which are much stronger to Europe given its colonial history.^50^

The biosafety frameworks in the EU and the selected SSA countries all have administrative systems and legal frameworks to guide the approval processes. The regulatory systems, however, continue to evolve independently in both EU and the selected countries. The influence attributed to the EU to the so-called “slow adoption” of GMOs in Africa is not reflected in the diverse laws as most countries refer to CBD and the CPB rather than mirroring EU Directives. Beyond reference to CBD, individual countries also differ in their respective characteristics, compositions, and workings. Even if EU have an influence in Africa, among others through the critical campaigns by several advocacy groups,^50^ evidence “on the ground” such as successful field trials and new commercialization of cowpea in Africa’s largest populated country, as well as the apparently more liberal approach to non-transgenic gene technology products, suggests otherwise. Field trials with GM crops have on numerous occasions been carried out in several African countries, without any recorded major hindrance. Hindrance is on the other hand reported toward commercial release, as observed by Komen and others.^39^ Recent approvals of GM cowpea, cotton and maize in Nigeria and GM cotton in Kenya, however, show that after a successful transfer of desired traits in preferred cultivars, commercialization can also take place.

Another form of possible EU-associated influence was encountered in Kenya where Kenya’s NBA, according to the interview conducted, reportedly declined to grant a biosafety permit for environmental release of *Gypsophila* cut flower despite applicants meeting all the biosafety requirements. The reason for declining was based on a possibility of jeopardizing the market share of the flower market in Europe based on SECs. This is corroborated in a 2022 Business Daily News Paper where the Chairman of the Kenya’s NBA says the refusal to grant the permit was purely socio- economic and not biosafety, a decision he further agrees was arrived at because of fear of losing the European flower market.^51^ There are also, however, reported political decisions made based on SECs or politics that are not directly linked to EU influence include the ban on importation of GMO foods into Kenya,^42^ and failure of Uganda’s president to assent to the country’s Biosafety Bill in 2017 and 2019.^52^ Cabinet in its decision in October 2022 lifted the ban on importations of GMO foods into Kenya,^43^ the lifting has, however, been challenged by Anti GMO activists in Kenya’s high court where an injunction was placed waiting hearing.^53^

### Opportunities for EU-SSA Plant Gene Technology Research Collaborations

Over the years, some African countries including Kenya, Nigeria and Uganda have developed capacity for R&D activities with plant gene technologies through external collaborations and support. To further strengthen this capacity as new biotechnology tools emerge, it is important that research collaboration are not faced with unnecessary barriers and costs.

The triangular collaboration being developed between the International Institute of Tropical agriculture (IITA), Nigeria, IITA Kenya, and the Swedish University of Agricultural Sciences (SLU), Sweden, is such as example. It has funding from the Swedish Research Council and focuses on the application of CRISPR/Cas editing for biofortification in cassava (Olayide, Alexandersson). The cassava biofortification project is part of an effort to reduce the incidence of vitamin A deficiency (VAD) in SSA. IITA Kenya was selected as the location for conducting the experiments because genome editing in cassava was already underway in this laboratory and this collaboration will allow for transfer of the knowledge to Nigeria. The approval to use CRISPR/Cas9 technology to improve the nutritional quality of cassava under contained use conditions from the National Biosafety Authority (NBA) in Kenya took approximately ten months. This was a long period of time, but the ability of IITA Kenya to genetically transform cassava by far outweighed the length of the approval process. We also wanted to promote a South-South collaboration and technology transfer. The total costs for running this project are approximately €5000/month, of which the approval costs were a smaller part and not a major hurdle. It should, however, be noticed that no field trials were planned for the biofortified cassava. Nevertheless, in a collaboration between the International Potato Center (CIP) in Kenya and SLU, which is also a part of the same project, field trials with late blight-resistant potato material from CIP have been carried out in Kenya and Sweden in parallel, followed by an exchange of data and discussion on the outcomes regarding the measured efficiency of acquired field resistance as well as societal perception and possibilities of future adaptation to commercial production.

For these collaborations to develop successfully, it is important that the biosafety regulatory regimes in the respective countries converge to an extent that promotes rather than inhibits technology transfer. In this respect, there are positive indications for the application of new techniques such as gene editing. Steps are being taken in Kenya, Nigeria, and Uganda to enable R&D activities with non-transgenic, gene-edited plants, whereas the current situation in Sweden is such that these activities (including field trials) are allowed under a GMO permit, which is easier than in many other European countries to attain.

Establishment of clear guidelines as in Nigeria, Kenya, and Uganda for determining whether gene-edited crops should go through rigorous biosafety procedure or not will facilitate quick response toward addressing existing crop challenges like late blight in potato, bacterial wilt in banana, as well as emerging challenges like fall armyworm. The approaches Nigeria, Kenya and Uganda have taken to handle gene edited crops in accordance with the presence or absence of a transgene(s) is already being practiced in the USA and Argentina, among others. This will likely contribute to creating a conducive environment for research collaborations A support of the “case-by-case” approach of handling gene-editing regulations and guidelines by regulators in the selected SSA countries and elsewhere in Africa could quicken the product development process as well as reducing research costs associated with biosafety regulations.

Our study describes the biosafety regulatory frameworks and their respective implementation in the selected countries and demonstrates that these are relatively straightforward and functional while not presenting any significant obstacles to R&D activities with gene technologies in plants. We provide a few examples, including our own research collaboration, as illustration. It is however important to follow up this study with a more extensive quantitative investigation, and with suitable comparators such as another group of countries that may experience a different regulatory situation, to be able to determine whether the respective regulatory frameworks have a stimulating or inhibitory effect on gene technology R&D. We nevertheless believe that the results of this study are important in particular for research institutes across SSA and EU in the preparations of research collaborations, and for policy and law makers in the selected countries as they can learn from the experiences and aim for a “best practices” approach.

## Supplementary Material

Supplemental Material
