# Supplementary material for: Biosafety regulatory frameworks in Kenya, Nigeria, Uganda and Sweden and their potential impact on international R&D collaborations
Source: GM Crops Food. 2023 Mar 28;14(1):1–17. doi: 10.1080/21645698.2023.2194221 (PMC10072116; doi:10.1080/21645698.2023.2194221)
Supplement: Supplemental Material [file KGMC_A_2194221_SM0760.docx]

Supplementary material

**Supplementary Information 1. List of regulators and researchers interviewed.**

*Regulators*

Dr. Charles Mugoya

Chairperson National Biosafety Committee (By time of interview)

Kampala, Uganda

[**mugoyac@gmail.com**](mailto:mugoyac@gmail.com)

Dr. Rufus Ebegba

Director General

National Biosafety Management Agency

Abuja, Nigeria

[**rebegba@gmail.com**](mailto:rebegba@gmail.com)

Prof. Theophilus M. Mutui (Phd)

Acting Director Technical Services,

National Biosafety Authority (By time of interview)

Nairobi, Kenya

[**mutui@biosafetyknya.go.ke**](mailto:mutui@biosafetyknya.go.ke)

*Researchers/Field Trial Managers*

Jude Aleu

Field Trial Manager

Virus Resistant Cassava for Africa Project, Uganda

Namulonge, Uganda

[**judealeu@yahoo.co.uk**](mailto:judealeu@yahoo.co.uk)

Ihouma Ikwonu (Phd)

Chief Research Officer

National Root Crops Research Institute, Umdike

Principle Investigator/Trial Manager,

Virus Resistant Cassava for Africa project, Nigeria

[**Ihouma.okwuonu@yahoo.com**](mailto:Ihouma.okwuonu@yahoo.com)

Hanington Obiero

Senior Research Officer,

Kenya Agricultural Livestock Research Organisation

Trial Manager,

Virus Resistant Cassava for Africa project, Kenya

[**hmobiero@yahoo.com**](mailto:hmobiero@yahoo.com)

Marit Lenman

Researcher

Department of Plant Protection Biology

Swedish University of Agricultural Sciences

Box 190, 234 22 Lomma, Sweden

[marit.lenman@slu.se](mailto:marit.lenman@slu.se)

**Supplementary Information 2. List of legal and policy documents reviewed in this study.**

*Kenya*

The Biosafety Act, 2009

<https://www.biosafetykenya.go.ke/Docs/Biosafety%20Act%202009(1).pdf>

The Biosafety (Contained Use) Regulations, 2011

<https://www.biosafetykenya.go.ke/Docs/The%20Biosafety%20(Contained%20Use)%20Regulations,%202011(1).pdf>

The Biosafety (Environmental Release) Regulations, 2011

<https://www.biosafetykenya.go.ke/Docs/The%20Biosafety%20(Environmental%20Release)%20Regulations,%202011(2).pdf>

The Biosafety (Import, Export and Transit) Regulations, 2011

<https://www.biosafetykenya.go.ke/Docs/The%20Biosafety%20(Import,%20Export%20and%20Transit)%20Regulations,%202011(1).pdf>

Approved Confined Field Trials (CFTs) Activities of Genetically Modified Organisms

<http://ke.biosafetyclearinghouse.net/approvedcft.shtml>

A Guide to Applicants on GMO Application Submission Timelines

<https://www.biosafetykenya.go.ke/images/A-GUIDE-TO-APPLICANTS-ON-GMO-APPLICATION-SUBMISSION-TIMELINES-1.pdf>

National Biosafety Authority (NBA) Fees, 2017

<https://infotradekenya.go.ke/media/Fees-Reviews%20NBA.pdf>

*Nigeria*

National Biosafety Management Agency Act, 2015

<https://nbma.gov.ng/wp-content/uploads/2021/04/National_Biosafety_Management_Agency__Act_2015-signed.pdf>

Amendment of the National Biosafety Management Agency Act, 2019.

<https://nbma.gov.ng/wp-content/uploads/2021/04/Amendment-of-NBMA-Act-2019.pdf>

National Guidelines for Institutional Biosafety Committee (IBC), 2018.

<https://nbma.gov.ng/wp-content/uploads/2021/04/NATIONAL-GUIDLINES-FOR-INSTITUTIONAL-BIOSAFETY-COMMITTEE-.pdf>

Fees for Various Biosafety Permits in Nigeria, 2017.

<https://nbma.gov.ng/wp-content/uploads/2021/04/Fees-and-Charges-for-Biosafety-Application-.pdf>

National Biosafety (Implementation, etc.) Regulations, 2017

<https://www.aatf-africa.org/wp-content/uploads/2021/02/Nigeria-Biosafety-Regulations-2017.pdf>

National Guidelines for the Regulation of Gene Editing, 2020

<https://nbma.gov.ng/wp-content/uploads/2021/04/NBMA-GENE-EDITING-GUIDELINE.pdf>

*Uganda*

Uganda National Council for Science and Technology Act, 1990

<https://old.ulii.org/ug/legislation/consolidated-act/209>

Confined Field Trial Guidelines for Uganda

<https://www.scifode-foundation.org/attachments/article/37/National%20Guidelines%20for%20Conduct%20of%20Confined%20Field%20Trials%20with%20GM%20crops.pdf>

National Biotechnology and Biosafety Policy, 2008

<http://extwprlegs1.fao.org/docs/pdf/uga204850.pdf>

*EU*

Directive 90/220/EEC of 23 April 1990 on the deliberate release into the environment of genetically modified organisms

<https://eur-lex.europa.eu/legal-content/EN/ALL/?uri=CELEX%3A31990L0220>

Directive 2001/18/EC of the European Parliament and of the Council of 12 March 2001 on the deliberate release into the environment of genetically modified organisms and repealing Council Directive 90/220/EEC - Commission Declaration

<https://eur-lex.europa.eu/legal-content/EN/TXT/?uri=CELEX:32001L0018>

Regulation (EC) No 1829/2003 of the European Parliament and the Council of 22 September 2003 on genetically modified food and feed

<https://eur-lex.europa.eu/legal-content/EN/TXT/PDF/?uri=CELEX:02003R1829-20080410&rid=1>

Regulation (EC) No 1830/2003 of the European Parliament and the Council of 22 September 2003 concerning the traceability and labeling of genetically modified organisms and the traceability of food and feed products produced from genetically modified organisms and amending Directive 2001/18/EC

<https://eur-lex.europa.eu/LexUriServ/LexUriServ.do?uri=OJ:L:2003:268:0024:0028:EN:PDF>

Directive (EU) 2015/412 of the European Parliament and of the Council of 11 March 2015 amending Directive 2001/18/EC as regards the possibility for the Member States to restrict or prohibit the cultivation of genetically modified organisms (GMOs) in their territory

<https://eur-lex.europa.eu/legal-content/EN/TXT/?uri=CELEX:32015L0412>

Court of Justice of the European Union PRESS RELEASE No 111/18 Luxembourg, 25 July 2018 Judgment in Case C-528/16 on Organisms obtained by Mutagenesis

<https://curia.europa.eu/jcms/upload/docs/application/pdf/2018-07/cp180111en.pdf>
